# O061. Frontal thermography in healthy individuals and headache patients: reliability of the method

**DOI:** 10.1186/1129-2377-16-S1-A122

**Published:** 2015-09-28

**Authors:** Cristina Voiticovschi-Iosob, Fabio Antonaci, Elena Rossi, Alfredo Costa, Ennio Pucci, Grazia Sances, Giorgio Dalla Volta

**Affiliations:** State Medical and Pharmaceutical University “Nicolae Testemitanu”, Chisinau, Moldova; University of Pavia, Pavia, Italy; Pavia Headache Science Centre, C. Mondino National Institute of Neurology Foundation, IRCCS, Department of Brain and Behavioral Sciences University of Pavia, Pavia, Italy; Politecnico di Milano, Dipartimento di Elettronica, Informazione e Bioingegneria, Milan, Italy; Istituto Clinico Città di Brescia, Centro Cefalee, Brescia, Italy

## Introduction

Infrared Thermography detects infrared lights emitted by the body to visualize changes in temperature due to abnormalities in the surface blood flow of affected areas. This method may aid in the diagnostic process in pain medicine.

## Objective

To assess the reliability of human body temperature measurement by means of Frontal Infrared Thermography (FIT).

## Methods

**Figure 1:**
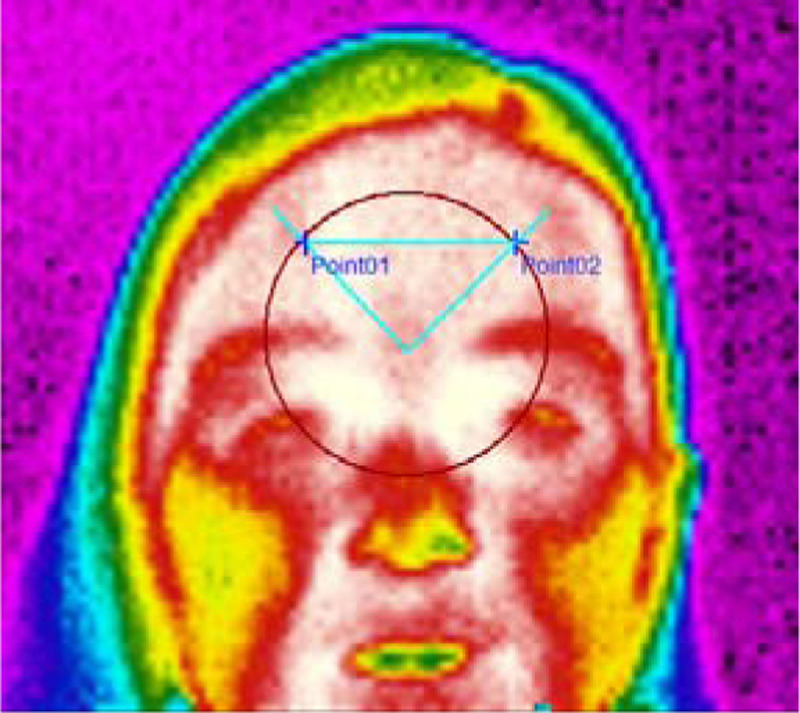
Standard evaluation obtained with infrared frontal thermography.

Thirty-five volunteers with a mean age of 35±11.6 years were evaluated. Fifteen of the 35 subjects were headache patients. FIT was assessed with an infrared thermal camera (model LT3, Zhejiang Dali Technology Co. Ldt) with a thermal sensitivity inferior than 0.08°C at 30°C. FIT measures the spatial distribution of the heat over the face and the image analysis evaluates the temperature in two target points (left and right side) in the frontal polar sites, equidistant 17 mm from Inion (fig. 1). The image analysis evaluated the temperature in two target points in the frontal polar sites. The measurements were performed in two separate sessions (T1 and T2), each session being the mean of three separate measurements. The Asymmetry Index, ANOVA 1 way, intra-class correlation coefficient and Pearson's correlation coefficient for the T1 were calculated. ANOVA 2 way compared the measurements between T1 and T2.

## Results

The analysis of variance did not show statistically significant difference between the three consecutive measurements during the first session (p = 0.21 right side; p = 0.35 left side) and the second session (p = 0.079 right side; p = 0.074 left side). Considering 0.75 the excellent reliability threshold (Fleiss), the measurements of both sides revealed a good reliability. The ICC values for right and left side were: 0.75 and 0.79 respectively during T1, and 0.72 and 0.74 respectively during T2. The best reliability was found between the second and the third measurement. The statistical test ANOVA 2 way did not reveal intra-individual test-retest variations. A low correlation (r ≤ 0.38) was found between FIT and external factors (room temperature, age and sex of subjects) while no correlation was found between FIT and pain side or VAS score.

## Conclusions

FIT can be an effective method for the temperature evaluation in humans. FIT measurements were symmetrical on both sides and were not influenced by room temperature, sex or age of the individuals. The Asymmetry Index was reliable in control subjects to describe the absence of lateralization of FIT and may be used as clinical control limits.

Written informed consent to publication was obtained from the patient(s).

